# Activation of EGFR/ERBB2 via Pathways Involving ERK1/2, P38 MAPK, AKT and FOXO Enhances Recovery of Diabetic Hearts from Ischemia-Reperfusion Injury

**DOI:** 10.1371/journal.pone.0039066

**Published:** 2012-06-13

**Authors:** Saghir Akhtar, Mariam H. M. Yousif, Bindu Chandrasekhar, Ibrahim F. Benter

**Affiliations:** Department of Pharmacology and Toxicology, Faculty of Medicine, Kuwait University, Kuwait City, Kuwait; University of Pecs Medical School, Hungary

## Abstract

This study characterized the effects of diabetes and/or ischemia on epidermal growth factor receptor, EGFR, and/or erbB2 signaling pathways on cardiac function. Isolated heart perfusion model of global ischemia was used to study the effect of chronic inhibition or acute activation of EGFR/erbB2 signaling on cardiac function in a rat model of type-1 diabetes. Induction of diabetes with streptozotocin impaired recovery of cardiac function (cardiac contractility and hemodynamics) following 40 minutes of global ischemia in isolated hearts. Chronic treatment with AG825 or AG1478, selective inhibitors of erbB2 and EGFR respectively, did not affect hyperglycemia but led to an exacerbation whereas acute administration of the EGFR ligand, epidermal growth factor (EGF), led to an improvement in cardiac recovery in diabetic hearts. Diabetes led to attenuated dimerization and phosphorylation of cardiac erbB2 and EGFR receptors that was associated with reduced signaling via extracellular-signal-regulated kinase 1/2 (ERK1/2), p38 mitogen activated protein (MAP) kinase and AKT (protein kinase B). Ischemia was also associated with reduced cardiac signaling via these molecules whereas EGF-treatment opposed diabetes and/or ischemia induced changes in ERK1/2, p38 MAP kinase, and AKT-FOXO signaling. Losartan treatment improved cardiac function in diabetes but also impaired EGFR phosphorylation in diabetic heart. Co-administration of EGF rescued Losartan-mediated reduction in EGFR phosphorylation and significantly improved cardiac recovery more than with either agent alone. EGFR/erbB2 signaling is an important cardiac survival pathway whose activation, particularly in diabetes, ischemia or following treatment with drugs that inhibit this cascade, significantly improves cardiac function. These findings may have clinical relevance particularly in the treatment of diabetes-induced cardiac dysfunction.

## Introduction

Diabetes significantly increases the risk of cardiovascular disease by 3- to 8-fold [1]. Current diabetic therapies are not sufficient to completely prevent development of diabetes-induced end-organ damage even if hyperglycemia is completely normalized [1]. Thus, it is becoming clear that signal transduction changes induced during hyperglycemia are not always reversed by current therapies designed to lower glucose levels and will also need to be normalized for effective treatment of diabetes complications. However, despite recent advances [2], the exact mechanisms leading to the development of cardiac dysfunction in diabetes and/or after ischemic injury are not fully understood.

The epidermal growth factor receptor (EGFR) family of receptor tyrosine kinases comprises four members: EGFR (erbB1), EGFR2 (erbB2, Neu, HER2), EGFR3 (erbB3) and EGFR4 (erbB4). Of these EGFR is a 175-kDa glycoprotein that can be activated by several different ligands including epidermal growth factor (EGF), heparin-binding EGF (HB-EGF), amphiregulin and betacellulin [3] to induce either homodimerization or heterodimerization with other EGFR family members, most notably erbB2 which is the preferred partner for dimerization. The erbB2 receptor lacks a ligand binding domain and therefore relies on dimerization with other EGFR family members for signaling. For example, EGF can serve as a ligand for activating EGFR and recruitment of erbB2; alternatively neuregulins (NRG) can serve as ligands for activating erbB4/erbB2 heterodimer signaling. Dimerization of erbBs results in subsequent phosphorylation of several downstream effector proteins including Ras, Raf, extracellular-signal-regulated kinase 1/2 (ERK1/2), p38 mitogen activated protein (MAP) kinase and phosphatidylinositol 3 (PI-3) kinase/AKT (protein kinase B) pathways [3]–[5]. Alternatively, EGFR transactivation can occur via G-protein coupled receptors (GPCR), such as angiotensin II (Ang II) and endothelin [6].

In experimental diabetes, upregulation of EGFR signaling as a result of increased gene expression and elevated receptor tyrosine kinase (RTK) activity leads to vascular dysfunction in several tissues and is therefore, detrimental in the vasculature whereas in the diabetic heart EGFR may have a beneficial role [7]–[10]. At least 3 out of the 4 erbB receptors, EGFR, erbB2, and erbB4, are detected in the adult human and rodent hearts [11]–[13] where they play an essential role in cardiac development during embryogenesis and might also be survival factors in the adult myocardium [14]–[17]. In the failing heart, the expression and activity of erbB2 and erbB4 receptors are depressed [18], [19] and signaling via erbB2/erbB4 heterodimers appears critical for adult cardiomyocyte survival [12], [20], [21]. The importance of erbB receptor signaling in normal cardiac physiology was not fully recognized until the unexpected and fatal cardiomyopathy reported in breast cancer patients [22], [23]. In patients receiving Trastuzamab, a monoclonal antibody inhibitor of erbB2, cardiac toxicity was noted in about 5% of patients receiving the antibody alone but this number increased to about 27% of patients when given in combination with anthracyclines [23], [24]. Surprisingly, cardiac toxicities were not always noted with other types of erbB receptor blockers implying that cardiac effects of erbB2 might be related to the specific drug used [23], [25], [26]. More recently, signaling through EGFR was shown to provide cardioprotection against stress-induced injury, and reduction in EGFR activity impacts on cardiomyocyte hypertrophy and survival [27].

**Table 1:** Effect of chronic treatment with AG1478 or AG825 on post ischemic recovery in cardiac contractility and coronary flow.

Groups studied	Pmax (mmHg)			LVEDP (mmHg)			+dp/dt (mmHg s^−1^)			−dp/dt (mmHg s^−1^)			Coronary Flow(ml min−1)		
	Baseline	REP	% R	Baseline	REP	% R	Baseline	REP	% R	Baseline	REP	% R	Baseline	REP	% R
Control ©	104±12	61±7	59±3	7.1±0.6	28±1	406±25	3770±323	2238±179	60±5	−2429±195	−1477±154	61±4	7.3±0.5	4.0±0.3	55±3
C+AG1478	88±5	38±4	43±4*	6.8±0.2	31±1	457±19*	2883±604	1330±270	46±2*	−1847±384	−808±175	43±1*	8.8±0.9	3.9±0.5	43±2*
C+AG825	73±6	24±2	33±2*	7.9±0.5	48±3	610±15*	2676±451	878±139	33±3*	−1670±339	−513±105	32±4*	6.9±0.2	2.0±0.1	29±2*
Diabetes (D)	75±15	10±2	14±1*	7.0±0.4	39±1	560±31*	3054±717	288±73	10±1*	−1791±450	−231±53	13±1*	8.3±1.0	1.0±0.1	12±1*
D+AG1478	61±4	5±1	8±1^#^	7.1±0.3	47±3	668±16^#^	2970±241	223±42	8±2	−1117±144	−156±16	14±1	6.6±0.2	0.7±0.1	11±1
D+AG825	83±10	6±2	7±1^#^	7.3±0.2	51±3	701±20^#^	2630±488	195±62	7±1^#^	−1507±367	−147±23	11±1	6.4±0.4	0.4±0.1	8±1^#^

The data for Baseline was computed at 30 min perfusion period before ischemia and the data for Reperfusion (REP) was computed at 30 min reperfusion period, and expressed as mean±SEM (N=6). Pmax=Left ventricular developed pressure; LVEDP=Left ventricular end-diastolic pressure; +dp/dt=Positive derivative of pressure; −dp/dt=Negative derivative of pressure; %R=% recovery=(reperfusion/baseline)×100;

* Value significantly different from Control, p<0.05;

# Value significantly different from Diabetes, p<0.05.

**Figure 1:**
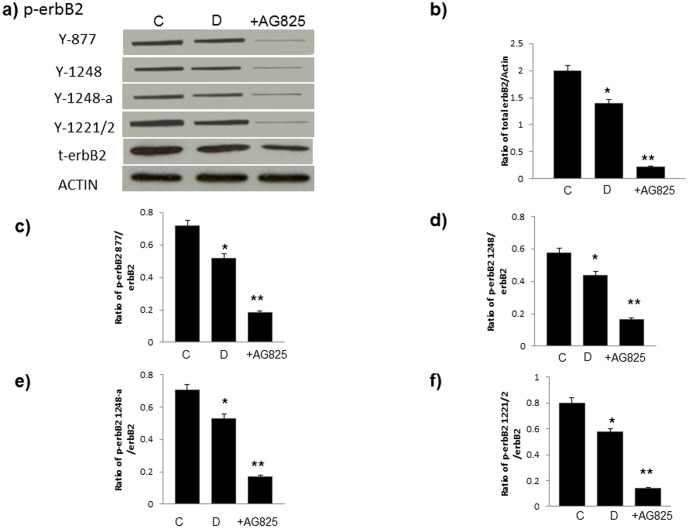
Diabetes is associated with reduced expression and phosphorylation of erbB2 receptor at multiple tyrosine sites that can be further inhibited by chronic treatment with AG825. **a**) Representative Western blots showing levels of phosphorylated erbB2 at Y877, Y1248, Y1248-a (which represents detection of Y1248 using an alternative antibody (p- erbB2-Antibody (Tyr1248)/EGFR (Tyr1173)) and Y12221/2 as well as total erbB2 (t-erbB2) and actin as a control protein in non-diabetic control hearts (C), diabetic hearts (D) and diabetic hearts chronically treated with AG825 (+AG825). **b**) quantification of erbB2 expression relative to actin and **c–f**) quantification of erbB2 phosphorylation at the stated tyrosine site relative to total erbB2 expression for all the groups studied by densitometry. N=4; * significantly different from control (p<0.05); ** significantly different from diabetes (p<0.05).

**Figure 2:**
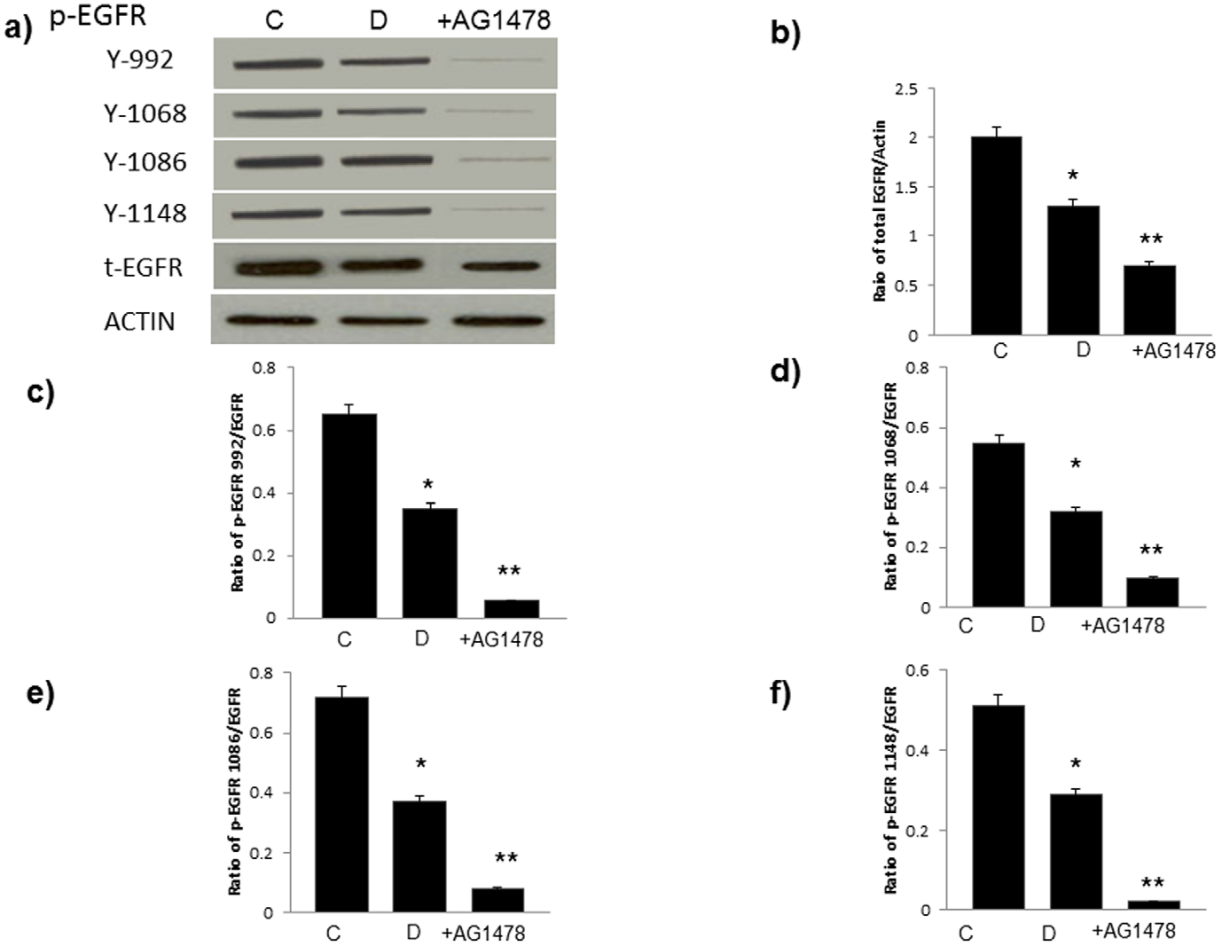
Diabetes is associated with reduced expression and phosphorylation of EGFR receptor at multiple tyrosine sites that can be further inhibited by chronic treatment with AG1478. **a**) Representative Western blots showing levels of phosphorylated EGFR at Y992, Y1068, Y1086, and Y1148 as well as total EGFR (t-EGFR) and Actin as a control protein in non-diabetic control hearts (C), diabetic hearts (D) and diabetic hearts chronically treated with AG1478 (+AG1478). **b**) quantification of EGFR expression relative to actin and **c–f**) quantification of EGFR phosphorylation at the stated tyrosine site relative to total EGFR expression for all the groups studied by densitometry. N=4; * significantly different from control (p<0.05); ** significantly different from diabetes (p<0.05).

**Figure 3:**
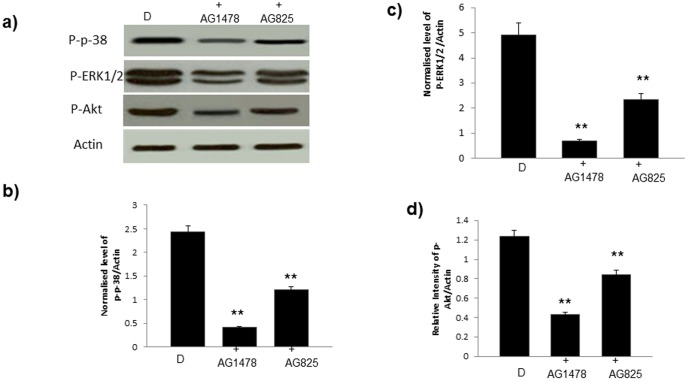
The effect of erbB inhbitors on phosphorylation of ERK1/2, p38 MAP kinase and AKT signaling. **a**) Representative Western blots and **b–d**) quantification of phosphorylation for the stated molecule relative to total actin expression for all the groups studied by densitometry. N=4; ** significantly different from diabetes (p<0.05).

**Figure 4:**
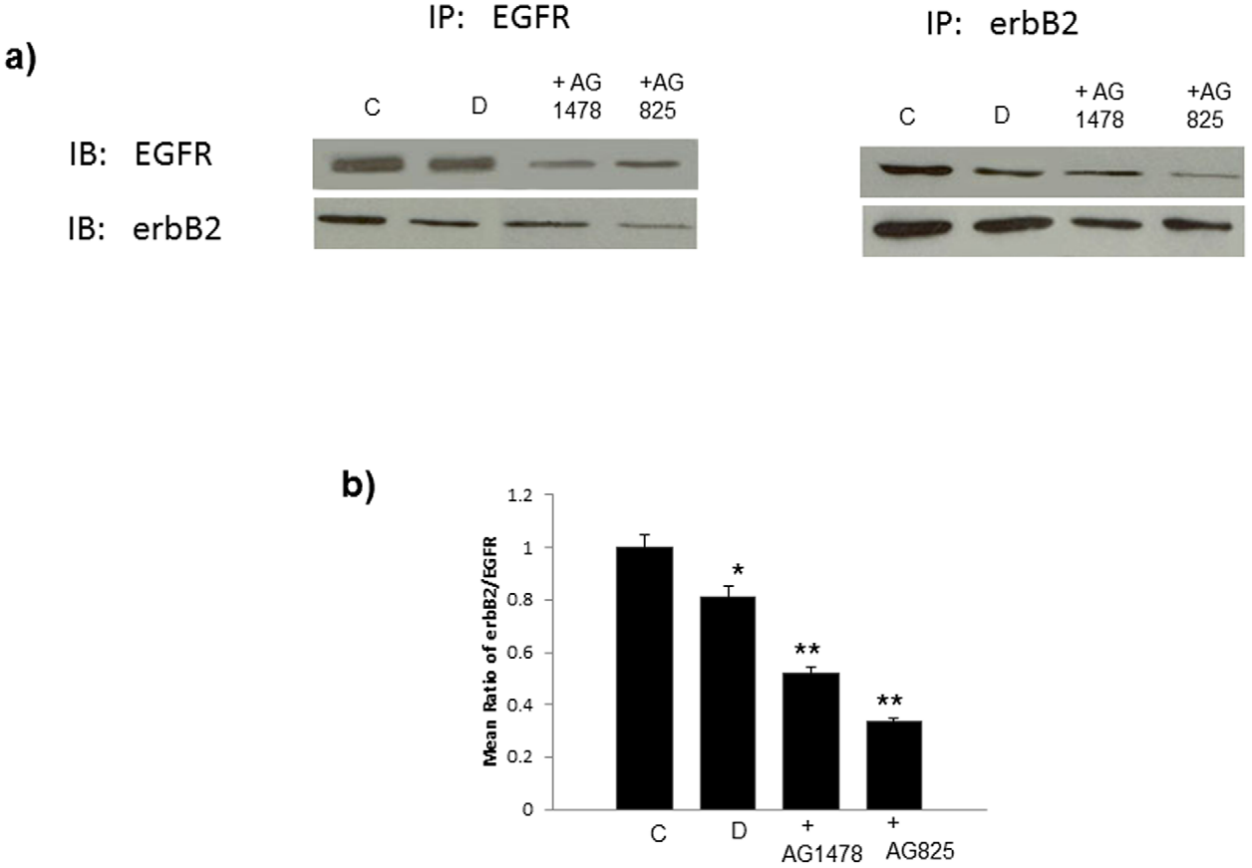
ErbB2/EGFR dimers are reduced in diabetes and by chronic treatment with AG1478 or AG825. Panel a) are represenatative Western Blots following immunoprecipitations (IP) with either total-EGFR or total-erbB2 antibody and subsequent immunoblotting (IB) with both antibodies individually. Panel b) represents the mean ratio of erbB2/EGFR dimers as assessed by densitometry for non-diabetic control hearts (C), diabetic hearts (D) and diabetic hearts chronically treated with AG1478 (+AG1478) or AG825 (+AG825). N=4; * significantly different from control (p<0.05); ** significantly different from diabetes (p<0.05).

**Figure 5:**
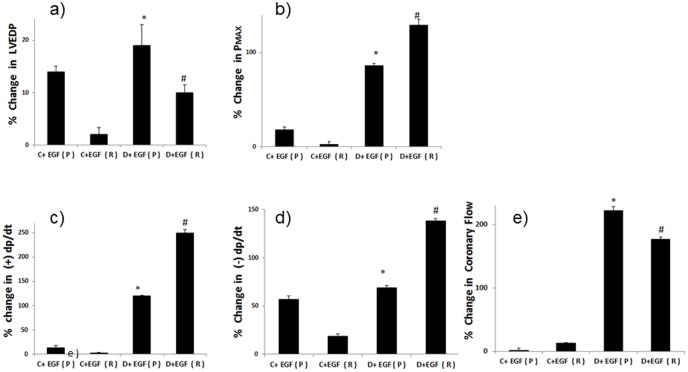
A comparison of the % change in cardiac function parameters for normal (C) and diabetic (D) hearts following acute EGF administration (+EGF) before ischemia (i.e. during perfusion (P)) or after ischemia (i.e. during reperfusion (R)). The percent change in parameter is calculated relative to the % recovery seen in the respective non-diabetic (C) or diabetic (D) controls and plotted for **a**) LVEDP; **b**) Pmax; **c**) +dp/dt; **d**) −dp/dt; and **e**) coronary flow. * significantly different from control (p<0.05); # significantly different from diabetes (p<0.05).

**Table 2:** Effect of Epidermal Growth Factor (EGF) on post ischemic recovery in cardiac contractility and coronary flow.

Groups studied	Pmax (mmHg)			LVEDP (mmHg)			+dp/dt (mmHg s^−1^)			−dp/dt (mmHg s^−1^)			Coronary Flow (ml min−1)		
	Baseline	REP	% R	Baseline	REP	% R	Baseline	REP	% R	Baseline	REP	% R	Baseline	REP	% R
Control (C)	53±8	26±4	50±2	6.8±0.4	16±2	243±29	1719±325	797±132	47±2	−1649±283	−602±91	37±3	8.5±0.2	4.0±1.0	46±7
C+ EGF (P)	85±19	51±6	59±3*	6.7±0.6	14±1	210±4	3142±452	1631±159	53±4	−2094±411	−1191±152	58±4*	9.8±1.5	4.6±0.4	47±3
C+ EGF (R)	74±5	38±2	51±3	6.3±0.3	16±1	248±9	3131±154	1485±84	47±2	−2142±99	−949±27	44±2*	12.7±0.7	6.6±0.3	52±1
Diabetes (D)	75±15	10±2	14±1*	6.5±0.4	56±1	861±50*	3054±717	287±73	10±1*	−1791±450	−231±53	13±1*	8.5±0.2	0.8±0.1	9±1*
D+ EGF (P)	115±13	30±3	26±2^#^	7.0±0.3	49±4	696±64^#^	3215±189	698±50	22±1^#^	−2317±223	−488±66	22±4^#^	7.6±0.5	2.3±0.6	29±6^#^
D+ EGF (R)	78±17	27±4	32±2^#^	6.7±0.2	50±1	746±49^#^	2122±137	762±196	35±7^#^	−1561±182	−479±41	31±2^#^	9.7±2.5	2.6±1.0	25±3^#^

The data for Baseline was computed at 30 min perfusion period before ischemia and the data for Reperfusion (REP) was computed at 30 min reperfusion period, and expressed as mean ±SEM (N=6); Pmax=Left ventricular developed pressure; LVEDP=Left ventricular end-diastolic pressure; +dp/dt=Positive derivative of pressure; −dp/dt=Negative derivative of pressure; %R=% recovery=(reperfusion/baseline)×100; P=drug given during Perfusion before Ischemia; R=drug given during Reperfusion after Ischemia;

* Value significantly different from Control, p<0.05;

# Value significantly different from Diabetes, p<0.05.

**Figure 6:**
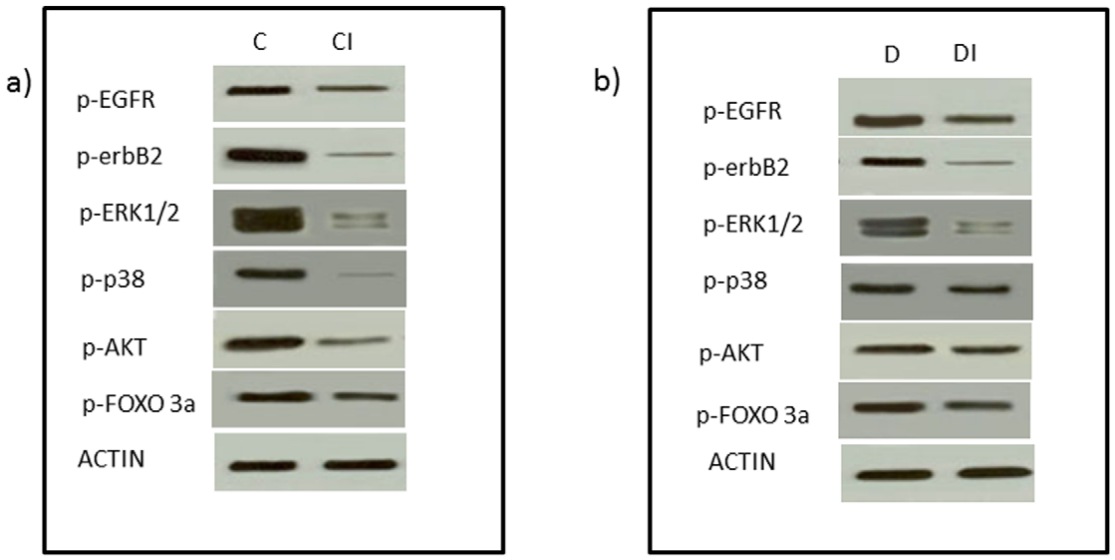
Representative Western blots to show ischemia-induced changes in phosphorylation of EGFR and erbB2 receptors and downstream signaling molecules for a) normal (non-diabetic) control hearts (C) and those subjected to 40 min ischemia (CI) and b) diabetic hearts (D) and those subjected to 40 min ischemia (DI).

**Figure 7:**
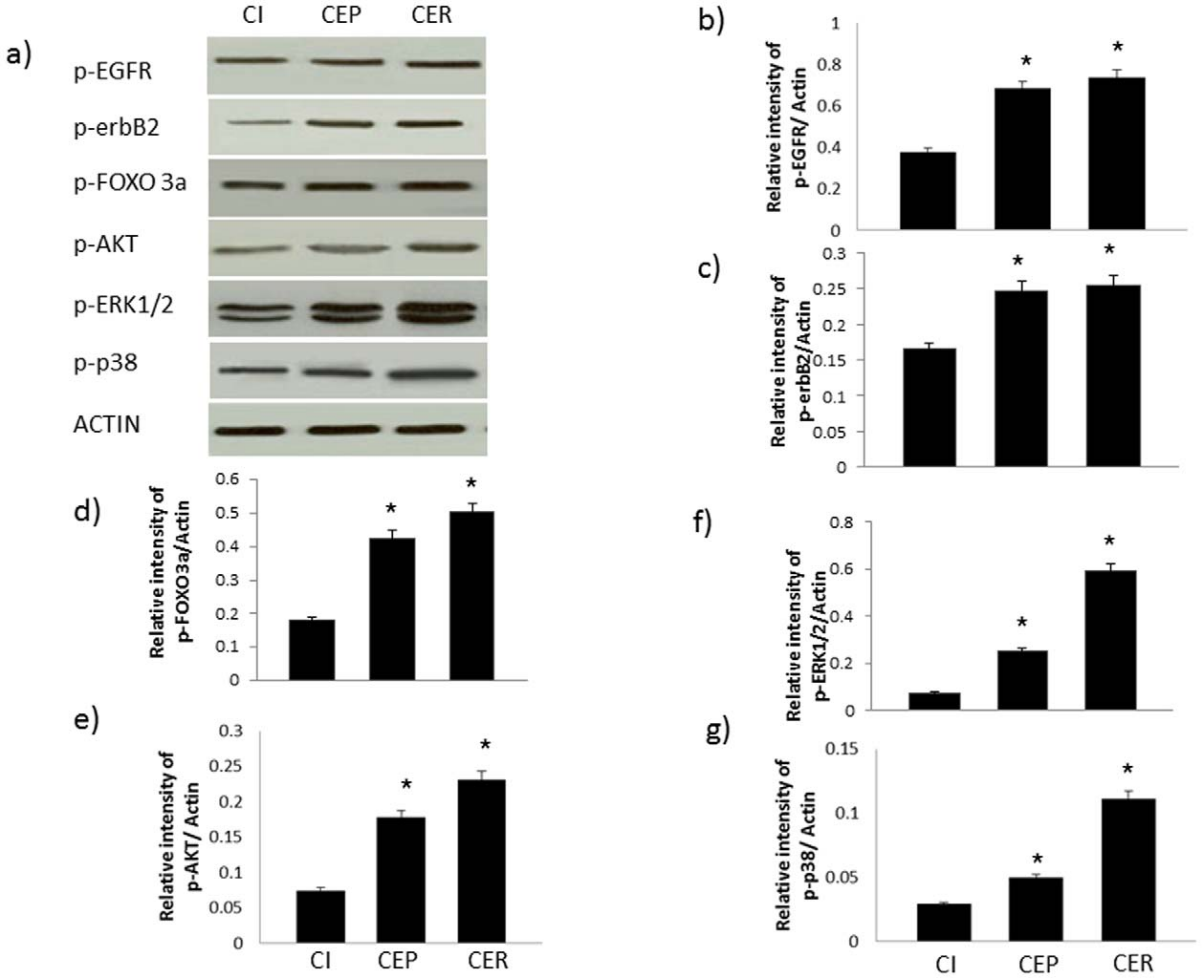
Acute EGF treatment in non-diabetic control hearts opposes the ischemia-induced changes in phosphorylation of EGFR/erbB2 signaling cascade. **a**) A representative Western blot of phosphorylation changes in key molecules following acute administration of EGF before ischemia (CEP) or after ischemia (CER) is compared to control hearts subjected to 40 mins ischemia (CI); b–g) densitometry plots quantifying the relative intensity of bands for the stated molecule relative to actin. N=4; * significantly different to CI.

**Figure 8:**
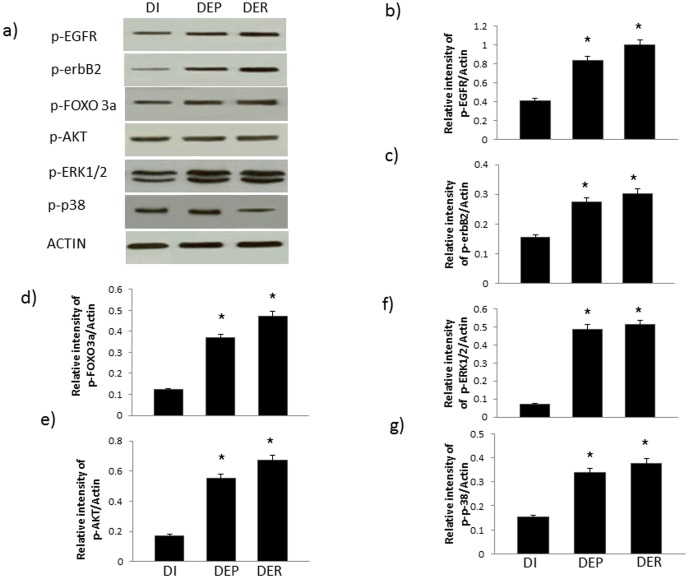
Acute EGF treatment in diabetic hearts opposes the diabetes and/or ischemia-induced changes in phosphorylation of EGFR/erbB2 signaling cascade. **a**) A representative Western blot of phosphorylation changes in key molecules following acute administration of EGF before ischemia (DEP) or after ischemia (DER) is compared to diabetic hearts subjected to 40 mins ischemia (DI); b–g) densitometry plots quantifying the relative intensity of bands for the stated molecule relative to actin. N=4; * significantly different to DI.

**Figure 9:**
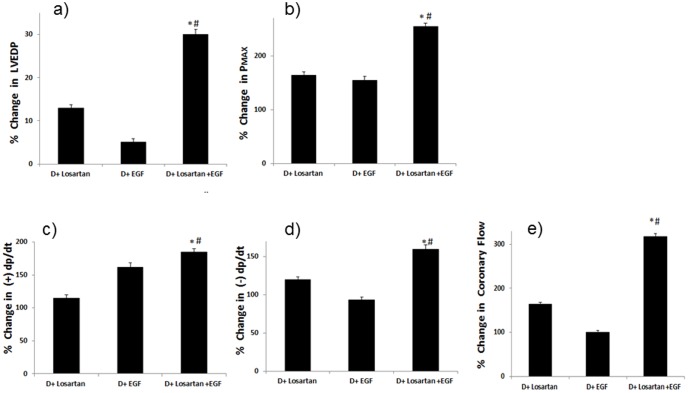
The % change in cardiac function parameters of diabetic (D) hearts treated with EGF (+EGF), Losartan (+Losartan) or their combination (+Losartan+EGF). The percent change in parameter is calculated relative to the % recovery seen in the respective diabetic (D) controls and plotted for **a**) LVEDP; **b**) Pmax; **c**) +dp/dt; **d**) −dp/dt; and **e**) coronary flow. * significantly different from D+ Losartan (p<0.05); # significantly different from D+ EGF (p<0.05).

**Table 3:** Effect of combined treatment with Losartan and Epidermal Growth Factor (EGF) on post ischemic recovery in cadiac contractility and coronary flow.

Groups studied	Pmax (mmHg)			LVEDP (mmHg)			+dp/dt (mmHg s^−1^)			−dp/dt (mmHg s^−1^)			Coronary Flow (ml min−1)		
	Baseline	REP	% R	Baseline	REP	% R	Baseline	REP	% R	Baseline	REP	% R	Baseline	REP	% R
Diabetes (D)	90±9	9±2	11±2	6.6±0.4	49±4	742±41	3166±655	377±92	13±2	−1737±403	−234±38	15±3	8.1±0.6	0.9±0.1	11±1
D+Losartan (R)	69±6	20±3	29±2*	6.6±0.1	43±1	645±12*	2127±105	591±129	28±5*	−1468±149	−485±96	33±3*	8.4±0.1	2.4±0.1	29±1*
D+EGF (R)	69±4	21±2	28±2*	6.5±0.3	45±1	692±52*	1743±244	609±155	34±6*	−1545±101	−448±70	29±4*	7.7±1.8	1.9±0.7	22±4*
D+Losartan+EGF (R)	80±6	31±3	39±1^#^	6.4±0.1	34±1	522±31^#^	2371±128	874±97	37±2^#^	−1814±30	−702±90	39±6	6.6±1.2	3.1±0.6	46±1^#^

The data for Baseline was computed at 30 min perfusion period before ischemia and the data for Reperfusion (REP) was computed at 30 min reperfusion period, and expressed as mean±SEM (N=6); Pmax=Left ventricular developed pressure; LVEDP=Left ventricular end-diastolic pressure; +dp/dt=Positive derivative of pressure; −dp/dt=Negative derivative of pressure; %R=% recovery=(reperfusion/baseline)×100; R=drug given during Reperfusion after Ischemia;

* Value significantly different from Diabetes, p<0.05;

# Value significantly different from D+Losartan, p<0.05.

**Figure 10:**
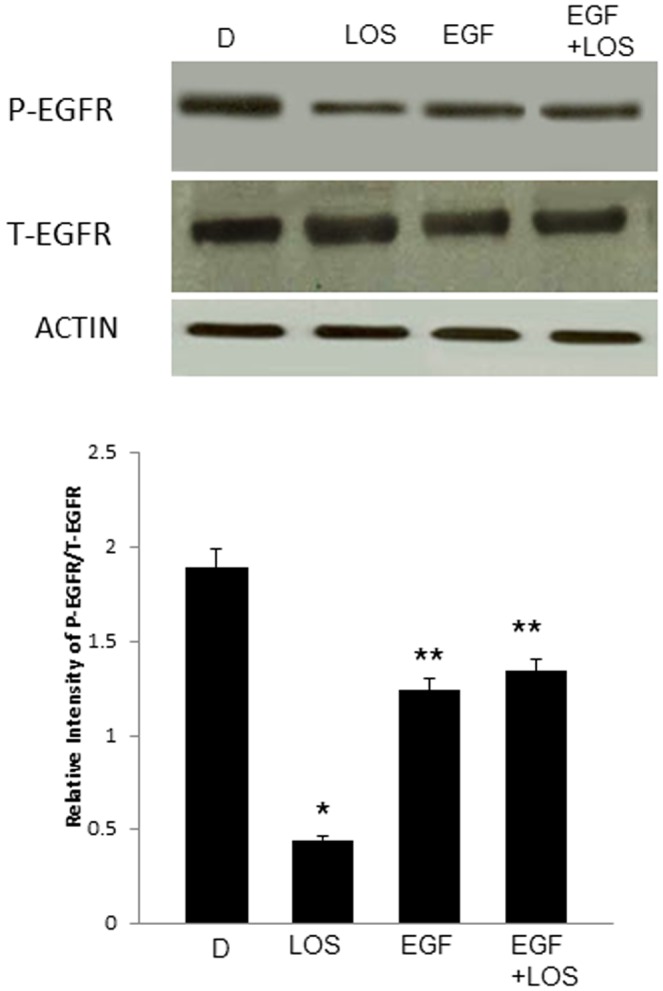
Acute EGF administration prevented Losartan-mediated inhibition of EGFR phosphorylation in diabetic hearts. **a**) A representative Western blot showing post-I/R levels of phosphorylated (p-EGFR) and total EGFR (T-EGFR) in diabetic hearts (D) and following acute administration during reperfusion of Losartan (LOS), EGF (EGF) or their combination (EGF+LOS) and b) densitometric histogram quantifying the relative intensity of p-EGFR/t-EGFR following normalization of each molecule to actin. N=4; * significantly different to D and ** significantly different to LOS.

**Figure 11:**
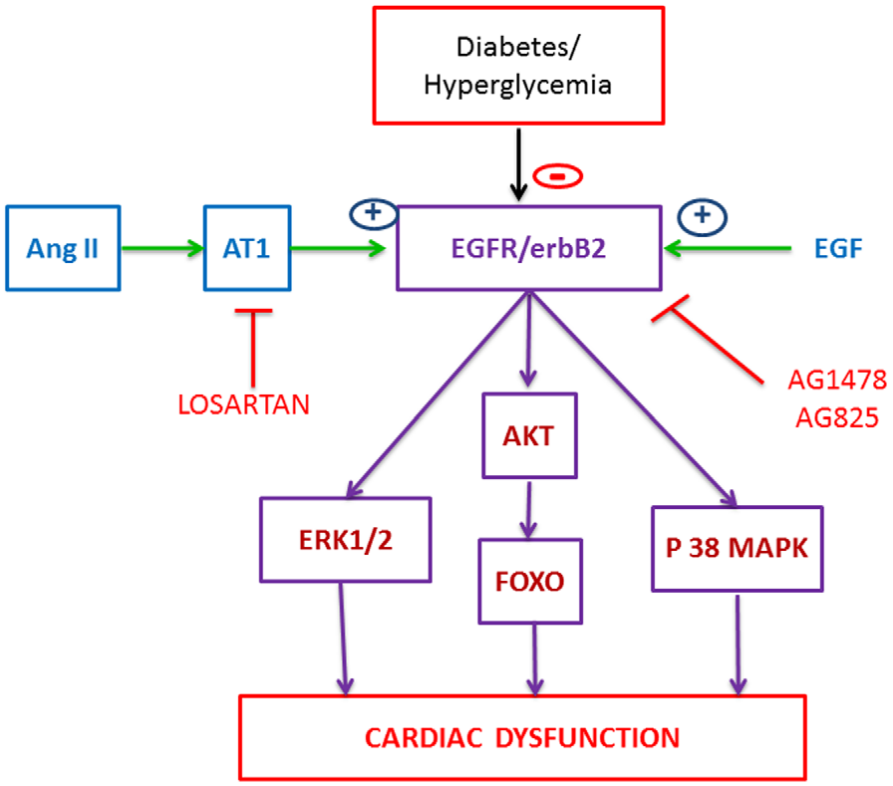
Schematic model summarising our findings on the role of EGFR/erbB2 signaling in diabetes-induced cardiac dysfunction. Diabetes and/or hyperglycemia via attenuation of the EGFR/erbB2 signaling and through subsequent modulation of downstream effectors such as ERK1/2, p38 MAPK or AKT/FOXO can lead to cardiac dysfunction. The effects of diabetes on EGFR/erbB2 pathway are exacerbated by blockade of this pathway by AG1478 or AG825 which leads to worsening cardiac recovery from I/R. However, the inhibitory effects of diabetes on EGFR/ErbB2 pathway may be opposed by administering EGF that also leads to improved cardiac function. The Angiotensin II (Ang II)/AT_1_ receptor pathway can also activate EGFR/erbB2 pathway that can be blocked by Losartan. Co-administration of EGF with Losartan attenuates losartan-mediated EGFR blockade and improves cardiac function in diabetes beyond that attained by either drug alone.

In the non-diabetic heart, we showed that chronic inhibition of EGFR with AG1478 attenuated the beneficial effects of cardiac preconditioning (PC) to ischemia-reperfusion injury implying that activation of EGFR signalling during PC is important for improving recovery following ischemia-reperfusion (I/R) injury [28]. However, the role of erbB receptor signaling in diabetes-induced cardiac dysfunction is not known. Thus in this study we sought to characterize the effects of inhibiting or activating EGFR or erbB2 signaling on cardiac function in isolated hearts subjected to I/R injury from normal or streptozotocin-induced diabetic rats.

FoxO transcription factors that belong to forkhead family of transcriptional regulators have diverse cellular functions including proliferation, apoptosis, DNA repair, autophagy and defense against oxidative stress depending on the cellular environment and tissue type ubiquitination [29]. In the heart, FoxO1 and FoxO3 proteins appear to be cardioprotective following oxidative stress through regulation of anti-oxidant gene expression and additional survival pathways [30]. The PI-3kinase/AKT pathway, downstream effector molecules of EGFR/erbB2 receptors, are major regulators of FoxO activity. PI-3 kinase-mediated phosphorylation of AKT leads to its nuclear localization and subsequent inactivation of FoxO via nuclear export of FoxO into the cytosol where it is degraded by ubiquitination [29]. Thus, we also sought to determine the changes in EGFR-erbB2/Akt-FoxO survival pathways after global ischemia and following activation of EGFR signaling by EGF in both normal and diabetic hearts.

## Methods

### Animal groups and drug treatment

All animal experiments in this study were approved by the Research Administration at Kuwait University and conformed to their ethics guidelines for the care and use of laboratory animals that are based on those published by the US National Institute of Health (NIH publication No 85-23, revised 1985).

### Induction of diabetes

Diabetes was induced by a single *ip* injection (55 mg/kg, body weight) of streptozotocin (STZ) in male Wistar rats weighing approximately 300 g as described previously [9], [10], [28]. Age-matched controls were injected with vehicle alone. The rats had free access to food and water throughout the study. Blood glucose levels were determined before and 48 hrs after injection of STZ. Rats with fasting blood glucose levels above 250 mg/dl were considered diabetic and included in the study. In addition, glucose levels of the rats were determined 4 weeks later prior to sacrificing the animals as described previously [31].

### Chronic treatments with EGFR and erbB2 inhibitors

The following animal groups were employed in this study. Group 1: Vehicle-treated (non-diabetic) control (C) animals. Group 2: AG825-treated control animals. Group 3: AG1478-treated control animals. Group 4: Vehicle-treated STZ-diabetic animals. Group 5: AG825-treated diabetic animals. Group 6: AG1478-treated diabetic animals.

AG825 or AG1478 at a dose of 1 mg/kg, or vehicle (1% DMSO in normal saline) was chronically administered *i.p.* every other day for four weeks starting from the day of diabetes induction. The dose of AG825 an AG1478 was selected based on our previous study in the same model of diabetes [9], [10]. N=10 animals per group.

### Acute administration of EGF and/or Losartan to isolated hearts from normal and diabetic rats

EGF (10^−8^ M) was infused into isolated hearts (from normal or diabetic rats) for a period of 10 min either immediately before ischemia (i.e. during perfusion) or for 10 min immediately after ischemia (i.e. during reperfusion). Losartan (2 µM) was infused for 10 min after ischemia either alone or in combination with EGF (10^−8^ M). The dose of EGF was selected based on previous animal studies [27], [32].

### Heart perfusion studies

The excised hearts were mounted on the Langendorff perfusion assembly (Hugo Sachs Electronics, Freiburg, Germany), and were perfused initially with a constant-pressure-perfusion of 50 mm Hg with oxygenated (95% O_2_ +5% CO_2_) Krebs – Henselit buffer (37°C) of the following composition (in mM): NaCl 117; KCl 4.39; CaCl_2_ 2.5; NaHCO_3_ 20.0; KH_2_PO_4_ 1.21; MgCl_2_.6H_2_O 1.2; Glucose 12.0; osmolarity 300 mOsm/l, pH 7.35. A water-filled balloon was introduced into the left ventricle and connected to a Statham pressure transducer (P23Db) and balloon volume was adjusted to give the baseline end-diastolic pressure of 5 mmHg. Perfusion pressure was measured immediately downstream from the flow probe in a branch of the aortic cannula using a Statham pressure transducer and was electronically maintained constant at 50 mmHg by means of a perfusion pressure control module. This system permits accurate adjustment of perfusion pressure between 5–300 mmHg to an accuracy of ±1 mmHg. Hearts were perfused for 30 minutes and then subjected to 40 minutes of ischemia (I) followed by a period of 30 minute reperfusion (R). Post-I/R cardiac parameters (Left ventricular developed pressure (Pmax), left ventricular end-diastolic pressure (LVEDP), ±dP/dT and coronary flow (CF) parameters were continuously recorded.

### ErbB receptor signaling studies

Western Blotting for total (t-) and/or phosphorylated (p-) forms of EGFR, erbB2, ERK1/2, p38 MAP kinases, AKT and FOXO3a were performed essentially as described by us previously [7], [8], [9], [33], [34]. Briefly, left ventricles of isolated hearts were dissected, snap-frozen in liquid nitrogen and stored at −80°C. The retrieved tissue samples were defrosted in ice, lysed and aliquots of equal protein subjected to SDS-PAGE gel electrophoresis and transferred onto PVDF membrane. Appropriate monoclonal and secondary antibodies (from either Upstate or Cell Signaling, USA) were used to detect phosphorylated and/or total forms of the desired proteins as described previously [7]. Actin was used as a loading control. Images were analysed and quantified by densitometry. The following antibodies from Cell Signaling (USA) were used in this study: t-EGFR-Antibody (rabbit) Cat. No. 2232, p-EGFR-Antibody (Tyr992) (rabbit) Cat. No. 2235, p-EGFR-Antibody (Tyr1068) (rabbit) Cat. No. 2234, p-EGFR-Antibody (Tyr1086) (rabbit) Cat. No. 2220, p-EGFR-Antibody (Tyr1148) (rabbit) Cat. No. 4404, t-Her2/ErbB2-Antibody (29D8) (rabbit) Cat. No. 2165, p-Her2/ErbB2-Antibody (Tyr877) (rabbit) Cat. No. 2241, p-Her2/ErbB2-Antibody (Tyr1248) (rabbit) Cat. No. 2247, p-Her2/ErbB2-Antibody (Tyr1248)/EGFR(Tyr1173) (rabbit) Cat. No. 2244, p-Her2/ErbB2-Antibody (Tyr1221/1222) (rabbit) Cat. No. 2243, p-Foxo3a (Ser 253) Cat. No. 9466, p-ERK1/2 (p44/42 MAP Kinase, Thr202/Tyr204) Antibody (rabbit) Cat. No. 9101. In addition the following antibodies were purchased from Santa Cruz, USA: p-Akt1/2/3 (Ser 473)-R Antibody: rabbit polyclonal IgG Cat. No. sc-7985-R and the p-p38 (Thr 180/Tyr182)-R rabbit polyclonal IgG K-2706 Cat. No. Sc-17852-R whereas the Anti-Actin rabbit polyclonal IgG (1 µl/10 ml) Cat. No. A-2066 was obtained from Sigma Chemical Co, USA.

### Receptor co-association immunoprecipitation studies

Anti-human EGFR (Cat. No. 2232; Cell Signaling, USA) or anti- human erbB2 antibody (Cat. No. 2165; Cell Signaling, USA) was added to 1 mg of tissue lysate sample at a dilution of 1∶50, and incubated on a tube rotator overnight at 4°C. Then 50 µl of protein A-agarose beads (Millipore, USA) was added to the samples and incubated on a end-over-end tube rotator at 4°C for a further 3 h. The bead pellets were then washed 3 times with excess lysis buffer containing protease inhibitor cocktail (Sigma, USA) (400 µl), and collected by centrifugation at the maximum speed for 5 seconds. The pellets were then resuspended in 30 µl of sample-loading buffer and and heated to 100°C for 10 min. Samples were subjected to SDS-PAGE electrophoresis and immnunoblotting was performed for EGFR or erbB2 as described above.

### Statistical analysis

Results were analyzed using Graph pad Prism software. Data are presented as mean ± SEM of ‘n’ number of experiments. Reperfusion values were compared with their respective baseline controls using a two tailed, paired *t*-test. Mean values were compared using analysis of variance followed by post hoc test (Bonferroni). The difference was considered to be significant when p value was less than 0.05.

## Results

### Blood Glucose levels

Induction of diabetes by STZ resulted in a significant increase in blood glucose concentration. Hyperglycemia persisted in the diabetic animals and was 593±13 mg/dl at four weeks as compared with 97±7 mg/dl in the control animals. Blood glucose levels were not significantly affected by chronic treatment with AG1478 (595±10 mg/dl) or AG825 (587±12 mg/dl) respectively.

### Cardiac contractility and hemodynamics in diabetic animals chronically treated with AG825 or AG1478

Table 1 shows the cardiac contractility parameters and coronary flow in normal or diabetic hearts treated with AG1478 (a selective inhibitor of EGFR tyrosine kinase activity) or AG825 (a selective inhibitor of erbB2 tyrosine kinase).

In isolated hearts subjected to 40 min of global ischemia followed by 30 mins of reperfusion, 4- weeks of diabetes led to a impaired recovery in all cardiac parameters studied compared to non-diabetic controls (Table 1). Chronic treatment with AG1478 or AG825 markedly reduced cardiac recovery in both normal and diabetes but the inhibitory effect appeared to be greater with AG825 (Table 1). In normal hearts, inhibitors had a much greater reduction in cardiac function than in diabetes. Diabetes alone resulted in a significant worsening of the recovery of hearts following I/R to an average % recovery of around 10%, and chronic treatment with AG1478 or AG825 of diabetic animals generally led to a further worsening in cardiac function beyond diabetes alone (Table 1).

### Diabetes is associated with reduced cardiac EGFR and erbB2 phosphorylation and attenuated downstream signalling via ERK1/2, p38 MAP kinase and AKT

Western blotting analyses showed that hearts isolated from STZ-induced diabetes, but not exposed to I/R, exhibited significantly reduced phosphorylation of EGFR and erbB2 at multiple tyrosine (Y) residues. In the case of erbB2 reduced phosphorylation was observed at Y877, Y1248 (as detected by two different primary antibodies) and Y1221/1222 (Figure 1) whereas EGFR phosphorylation was reduced at Y992, Y1068, Y1086, and Y1148 (Figure 2). Furthermore, chronic treatment of diabetic animals with either AG1478 or AG825 resulted in a significantly marked further inhibition in the phosphorylation of EGFR or erbB2 respectively at all the tyrosine residues studied (Figure 1 and 2). Both AG1478 and AG825 significantly inhibited phosphorylation of the potential downstream effectors, ERK1/2, p38 MAP kinase and AKT in diabetic hearts not exposed to I/R (Figure 3).

### Co-association of erbB2/EGFR receptors in diabetic and treated hearts

Immunoprecipitation studies were undertaken in all animal groups to ascertain whether EGFR and erbB2 could be co-precipitated – an indicator of their co-association or heterodimerization. Immunopreciptation of EGFR with an a specific antibody for EGFR resulted in co-precipitation of erbB2 and likewise, pull down of erbB2 with an anti-erbB2 antibody showed a co-association with EGFR (see Figure 4). The level of co-association of the two erbB receptors was reduced in diabetic hearts compared to normal. Also treatments with AG1478 or AG825 both reduced the levels of co-association of the two erbB receptors though the inhibition was greater with AG825 (see Figure 4).

### Acute administration of EGF before or after ischemia improves cardiac recovery in diabetic hearts

Acute administration of EGF, a ligand for EGFR, before ischemia or after ischemia led to a marked and significant improvement in cardiac contractility and coronary flow in diabetic hearts but little or only modest improvements in the cardiac function of normal hearts (Table 2). The effect of EGF in diabetic hearts was greatest for CF and minimal in the case of LVEDP (Table 2). Figure 5 highlights the relative % change in the various cardiac parameters between normal and diabetic hearts upon EGF administration either before or after ischemia. Administering EGF to diabetic hearts before ischemia appeared to yield a greater improvement in recovery of LVEDP and coronary flow whereas administration after ischemia led to a greater improvement in Pmax, +dp/dt, and −dp/dt (Table 2; Figure 5).

### Acute administration of EGF opposes ischemia-induced changes in phosphorylation of EGFR, erbB2, and signalling via ERK1/2, p38 MAP kinase, AKT and FOXO in diabetic hearts

Phosphorylation of EGFR, erbB2, ERK1/2, p38 MAP kinase, AKT and FOXO3a was reduced following 40 mins of ischemia in normal and diabetic hearts (Figure 6). In contrast, EGF administration either before or after ischemia enhances phosphorylation of EGFR, erbB2, ERK1/2, p38 MAP kinase, AKT and FOXO3a proteins in both normal (Figure 7) and diabetic hearts (Figure 8).

### Combined administration of EGF with Losartan improves cardiac recovery more than with each drug alone in diabetic hearts

We next compared the functional recovery of diabetic hearts treated with EGF with those treated with Losartan, an AT_1_ receptor blocker. EGF administration after ischemia at a concentration of 10^−8^ M resulted in comparable improvement in cardiac parameters compared with Losartan administered at a concentration of 2×10^−6^ M (Table 3). Further, combination treatment of hearts with both EGF and Losartan given simultaneously during reperfusion significantly enhanced recovery in all cardiac parameters more than with each drug alone (Table 3). In the case of % recovery in CF, the EGF and Losartan combination appeared to be additive (Table 3 and Figure 9). Combined EGF/Losartan-mediated improvement in +dp/dt was significantly higher than Losartan alone but not significantly different from EGF alone (Table 3 and Figure 9c).

Since Losartan is known inhibitor of Angiotensin II-mediated EGFR transactivation [6], we next examined the impact of Losartan and/or EGF administration on EGFR phosphorylation in diabetic hearts. Diabetic hearts treated with Losartan exhibited a lower EGFR phosphorylation compared to untreated diabetes following I/R. In contrast, combination treatment of Losartan with EGF, attenuated the Losartan-mediated reduction in EGFR phosphorylation where EGFR phosphorylation levels were elevated comparable to that of EGF alone (Figure 10).

## Discussion

The major novel finding of this study is that diabetes is associated with a reduction in EGFR/erbB2 heterodimer signaling in the heart and that activation of EGFR/erbB2 signaling via EGF leads to markedly improved cardiac recovery from I/R by opposing diabetes and/or ischemia induced changes in ERK1/2, p38 MAP kinase, AKT and FOXO signaling. In the diabetic hearts, this study showed that the relative improvements in cardiac function with EGF were similar to those observed with a clinically established AT_1_ receptor antagonist, Losartan. Further, since Losartan is known to also inhibit EGFR transactivation [6], [7], combination therapy with EGF significantly improved cardiac recovery more than with each drug alone. Losartan-induced reduction in EGFR phosphorylation was prevented by co-administration of EGF implying that rescuing the EGFR inhibitory effect of AT_1_ receptor antagonists by co-treatment with EGF may represent a novel clinical approach to improving cardiac function in diabetic patients.

In this study our initial goal was to determine whether the inhibition or activation of erbB2 and EGFR signaling was able to aid or exacerbate recovery of cardiac function following global ischemia in isolated hearts from normal or streptozotocin-induced diabetic rats. We found that chronic inhibition of either EGFR or erbB2 receptor signaling generally exacerbated recovery from ischemia-reperfusion injury of hearts isolated from normal and diabetes (Figures 1 and 2). However, the extent of cardiac dysfunction-induced by the inhibitors (Table 1) appeared to be greater in normal compared to diabetic hearts implying that the EGFR/erbB2 pro-survival pathway was significantly impaired in diabetes. Signaling studies confirmed that diabetes is associated with a reduction in EGFR and erbB2 phosphorylation at multiple tyrosine residues that appear to be important signalling cues for downstream effectors such as ERK1/2, p38MAP kinase and AKT (Figures 1 and 2). The fact that inhibition of erbB2 or EGFR signalling by AG825 and AG1478 respectively, in the diabetic heart also led to decreased phosphorylation of their likely downstream effectors ERK1/2, p38MAP kinase and AKT (see Figure 3) implied that these signalling molecules are common downstream effectors for EGFR and erbB signalling which might arise from a dimerization of these two erbB receptors. Figure 4 provided evidence for the likely dimerization or EGFR and erbB2 in normal and diabetic hearts. Importantly as implied by the cardiac function studies, compared to normal animals diabetes led to decreased levels of EGFR/erbB2 dimerization in the heart that was further reduced upon chronic treatment of animals with either AG1478 or AG825. The reduced dimerization may partly be explained by reduced expression of the two receptors in diabetic hearts as seen in Figures 1 and 2. Thus, our data suggest that EGFR and erbB2 heterodimerization-mediated signalling via ERK1/2, p38 MAP kinase and AKT pathways is at play in diabetic hearts which when chronically inhibited by either AG1478 or AG825 leads to worsening recovery of hearts from acute ischemia-reperfusion injury.

We next showed that activation of this pathway by acute administration of EGF significantly reversed the detrimental functional and biochemical changes following ischemic injury in diabetic hearts. Acute administration of EGF either before or after ischemia led to marked improvements in cardiac function in the diabetic heart following I/R (Table 2). The EGF-mediated recovery in cardiac parameters following I/R was generally much greater in diabetes than in normal hearts (Figure 5) implying the differential responsiveness of this pathway to ligand-mediated activation in the healthy and diseased heart. This is not an unexpected result as the normal heart already has a higher level of EGFR/erbB2 signaling compared to the diabetic heart where signaling is severely compromised (see Figures 1 and 2). A further point of note is that giving EGF after ischemia, rather than before ischemia, was equally or more effective in improving cardiac recovery following I/R (Table 2 and Figure 5) thereby implying that EGF administration even after an ischemic event may represent a novel therapeutic strategy for improving cardiac recovery in patients with diabetes.

Our findings that chronic inhibition of EGFR or erbB2 signaling markedly reduces cardiac function recovery following acute I/R injury in the normal rat heart are supported by other studies where either chronic [35] or acute [36] pharmacological inhibition in non-diabetic mice also led to similar conclusions as well as by recent studies where even acute activation of these pathways appeared protective in hearts subjected to ischemic stress [32]. Also chronic pharmacological inhibition of EGFR with AG1478 or EKB-569 (an irreversible tyrosine kinase inhibitor) or expression of mutant EGFR or erbB2 led to the development of cardiac dysfunction in non-diabetic hearts [35]. Here we presented evidence that further suggests that these pathways are even more important in diabetes than in normal hearts, a fact that might be clinically significant. It is already known that cardiac sensitivity of diabetic patients to some conventional antidiabetic drugs makes determining the specific treatment for diabetes-and its cardiac complications more difficult and there is a drive to identify novel drugs that do not impinge on cardiac function [37]. Our study highlights that drugs that likely directly or indirectly inhibit erbB receptor family signalling might more adversely affect cardiac function and/or cardiac recovery following ischemia. Thus, diabetic patients might be more sensitive to anticancer therapies directed at erbB receptors some of which are known to induce severe cardiac toxicities [23], [25], [26].

Of the relative contribution of erbB receptor signalling, erbB2 signaling appears to be more critical to cardiac recovery in both normal and diabetic hearts. This was evidenced from Figures 2 and 3 which show that at the doses used, AG825 or AG1478 gave similar inhibitions of erbB2 or EGFR phosphorylation respectively, but cardiac recovery in all the parameters studied was generally worse with AG825 (Table 1). This could also mean that in addition to signalling via erbB2/EGFR heterodimers, signalling via erbB2/erbB4 might also be involved, though this was not studied further here. Nonetheless, both signalling of erbB2 with erbB4 and EGFR appears to be important in the normal adult heart [27], [32].

Since the impact of cardiac ischemia on phosphorylation of EGFR and ERB2 as well as their downstream signaling molecules is not known, we next showed that hearts subjected to ischemia led to a reduction in EGFR and erbB2 phosphorylation as well as that of the known downstream effectors, ERK1/2 and p38 MAP kinases (Figure 6). Additionally, as the AKT/FOXO survival pathway has recently been shown to be an important survival signal in ischemia-reperfusion injury [38], we also investigated the effect of ischemia and EGF administration on this pathway in normal and diabetic hearts (Figures 6–8). Although other pathways can reportedly regulate FOXO activity [38], [39], the PI-3 kinase/AKT pathway appears to be important in the heart where it negatively regulates FOXO activity by increasing FOXO phosphorylation thereby leading to its inactivation via nuclear exclusion, polyubiquitination and degradation [29], [38]. PI3-kinase/AKT is a known downstream effector pathway of EGFR and erbB2 signaling. Consistent with this, we showed that diabetes-led to an attenuation in EGFR/erbB2/AKT pathway that was further attenuated upon exposure of hearts to ischemia and correlated with a worsening recovery of cardiac function following I/R (Table 2 and Figures 1, 2 and 6). In both normal and diabetic hearts, ischemia led to decreased phosphorylation of both AKT and FOXO3a at Ser ^253^ (Figure 6) whereas acute administration of EGF, either before or after ischemia, opposed these ischemia-induced changes in the AKT/FOXO3a pathway (Figures 7 and 8). Thus our data are consistent with the notion that ischemia leads to reduced EGFR/erbB2/AKT signaling that is coupled with a transient activation of FOXO signaling (represented by decreased phosphorylation at Ser ^253^) most likely as a compensatory mechanism to enhance cardiac survival pathways and inhibit pro-death signaling. However, this transient FOXO activation appeared to be insufficient in preventing ischemia-induced cardiac impairment as cardiac function was actually compromised following I/R. A recent study in cardiomyocytes is supportive of our finding where hypoxia led to significant decreases in FOXO protein phosphorylation consistent with its activation and nuclear localization [30]. Interestingly, administration of EGF, a specific ligand for EGFR, led to raised levels of phosphorylated EGFR and that of the ligand-less erbB2 receptor (Figures 7 and 8), implying the latter's recruitment into EGFR/erbB2 heterodimers. This is not an unexpected finding as we showed above (Figure 4) that diabetes is associated with reduced EGFR/erbB2 heterodimers and thus appear to be important signaling partners in cardiac function including following I/R injury. Further, EGF-mediated activation of EGFR/erbB2/AKT signaling in the hearts via modulation of FOXO3a activity appears to be a critical survival pathway in preventing diabetes-mediated cardiac dysfunction. However, whether these survival pathways are largely at play within the coronary vasculature and/or cardiac muscle is not known and requires further study.

Angiotensin II receptor blockers (ARBs) such as Losartan are clinically established cardioprotective agents in the treatment of diabetes-induced end-organ damage. However, since Losartan can also inhibit EGFR transactivation [6], [7], we hypothesized that the existing cardioprotective effects of ARBs might not be optimal and could be improved by preventing their inhibition of the pro-survival EGFR pathway. Indeed, our results showed that combination of Losartan with EGF administration after ischemia significantly improved cardiac recovery more than with each drug alone (Table 3 and Figure 9). Further, consistent with ARBs known inhibition of Angiotensin II-mediated transactivation of EGFR, diabetic hearts treated with Losartan reduced EGFR phosphorylation compared to untreated diabetes following I/R (Figure 10). In contrast, combination treatment of Losartan with EGF, prevented the inhibitory effects of Losartan on EGFR transactivation as phosphorylation levels were raised to similar levels as EGF administration alone (Figure 10). A schematic model summarising our findings on the role of EGFR/erbB2 signaling in diabetes-induced cardiac dysfunction is given in Figure 11. Our data may have important clinical implications as they suggest for the first time that rescuing the EGFR inhibitory effect of AT_1_ receptor antagonists or diabetes and/or ischemia by activators of the EGF/EGFR pathway may represent a novel clinical approach to improve protection against end-organ damage in diabetic hearts. However, the clinical success of such a strategy may require targeted cardiac delivery of EGF or its analogues as ligands for erbB receptors can have other, sometimes unwanted opposing, effects on different organ systems [9], [10], [40].
